# Recombinant Human Leptin Does Not Alter Gut Hormone Levels after Gastric Bypass but May Attenuate Sweet Cravings

**DOI:** 10.1155/2014/120286

**Published:** 2014-03-20

**Authors:** Rushika Conroy, Gerardo Febres, Donald J. McMahon, Michael O. Thorner, Bruce D. Gaylinn, Irene Conwell, Louis Aronne, Judith Korner

**Affiliations:** ^1^Division of Pediatric Endocrinology, Columbia University Medical Center, New York, NY 10032, USA; ^2^Division of Pediatric Endocrinology, Baystate Medical Center, Springfield, MA 01199, USA; ^3^Division of Endocrinology, Columbia University Medical Center, New York, NY 10032, USA; ^4^Division of Medicine, University of Virginia Medical Center, Charlottesville, VA 22902, USA; ^5^Division of Medicine, Cornell University Medical Center, New York, NY 10021, USA

## Abstract

Bariatric surgery improves glucose homeostasis and alters gut hormones partly independent of weight loss. Leptin plays a role in these processes; levels are decreased following bariatric surgery, creating a relative leptin insufficiency. We previously showed that leptin administration in a weight-reduced state after Roux-en-Y gastric bypass (RYGB) caused no further weight loss. Here, we discuss the impact of leptin administration on gut hormones, glucostasis, and appetite. Weight stable women after RYGB were randomized to receive placebo or recombinant human metreleptin (0.05 mg/kg twice daily). At weeks 0 and 16, a liquid meal challenge was performed. Glucose, insulin, C-peptide, GLP-1, PYY, glucagon, and ghrelin (total, acyl, and desacyl) were measured fasting and postprandially. Appetite was assessed using a visual analog scale. Mean post-op period was 53 ± 2.3 months; mean BMI was 34.6 ± 0.2 kg/m^2^. At 16 weeks, there was no significant change in weight within or between groups. Fasting PYY was significantly different between groups and the leptin group had lower sweets craving at week 16 than the placebo group (*P* < 0.05). No other differences were observed. Leptin replacement does not alter gut hormones or glucostasis but may diminish sweet cravings compared to placebo in this population of post-RYGB women.

## 1. Introduction

Roux-en-Y gastric bypass (RYGB) surgery results in a reduction of approximately 38% of total body weight at one year that, unlike diet therapy alone, is mostly maintained over the long term [1]. In addition to weight reduction, improvement in glucose homeostasis has also been observed, which may be partly independent of reduced body weight. Unique alterations in circulating levels of gut hormones, such as ghrelin, peptide YY (PYY), and glucagon-like peptide 1 (GLP-1), also occur after RYGB that create an environment favoring decreased appetite, weight reduction, maintenance of a reduced body weight, and improved glucose tolerance. Ghrelin, an orexigenic hormone produced in cells of the oxyntic glands of the stomach, was found to decrease or remain the same following RYGB [2, 3], in contrast to the usual increase in ghrelin levels that occurs after diet or gastric banding. PYY is secreted by intestinal L cells in response to food intake, leading to a decrease in gastrointestinal motility and increased satiety. Postprandial PYY levels are markedly increased after RYGB [2, 4]. Circulating concentrations of GLP-1, also produced by L cells, are increased following RYGB, contributing directly to reduced appetite, increased satiety, and weight loss as well as increases in glucose-stimulated insulin release following food ingestion [4, 5].

Many individuals who have undergone RYGB experience a plateau in weight loss with a body mass index (BMI) still within the obese range [6]. Counterregulatory hormones may impede further loss despite the presence of excess of body fat [7]. Leptin is a critical afferent component of a regulatory loop linking fat mass to food intake and energy expenditure and has also been shown to play an important role in glucose homeostasis through its effects on insulin as well as other mediators of glucose metabolism [8, 9]. Following weight loss, leptin levels decrease out of proportion to the amount of fat mass [10]. Leptin levels in those having lost weight following RYGB are less than levels in BMI-matched individuals who have not undergone weight loss [11], putting the former in a state of relative leptin insufficiency, which may be an important factor contributing to their inability to lose more weight.

Leptin is thought to modulate a number of hormones involved in appetite regulation and food metabolism, which are themselves altered by weight loss [12, 13]. While its relationship with some appetitive hormones is as yet unclear, animal studies have suggested that GLP-1 as well as PYY are increased following leptin administration [14–16], favoring appetite reduction and weight loss. Leptin and ghrelin have opposing actions and leptin administration in animal models has resulted in a reduction in ghrelin levels [17, 18]. Leptin administration has been shown to increase satiety and satiation in mouse models of obesity as well as in humans with leptin insufficiency [19–21], suggesting that it may affect the secretion and/or function of such hormones to promote weight reduction. Insulin sensitivity is improved following leptin administration [22], and leptin has been found to decrease glucagon levels in rat and mouse models of both type 1 and type 2 diabetes, contributing to the improvement in glycemic status [23, 24].

Leptin replacement therapy has been used in humans with congenital leptin deficiency and has resulted in weight loss when prescribed in physiologic doses. However, high pharmacologic levels of leptin are required to induce weight loss in otherwise healthy obese individuals; physiologic replacement of leptin has led to minimal to no weight loss [25–31]. In contrast, administration of physiologic replacement doses of leptin that restore circulating concentrations to preweight loss levels reverses many of the manifestations characteristic of the weight-reduced state, in some cases, irrespective of further weight loss [25–27]. Animal models of weight loss have suggested that leptin interacts with appetitive hormones in a manner that promotes further weight reduction [14, 15, 18]. Such interactions have yet to be studied in humans after RYGB.

We previously reported that, contrary to our expectation, leptin administration did not lead to further weight loss in women who had undergone weight loss after RYGB and whose leptin levels were lower than that predicted for a nonreduced individual with the same BMI [32]. This paper examines our secondary objective, which was to establish whether leptin administration in this weight-reduced state would be associated with changes in hormones involved in nutrient metabolism as well as in satiation. We hypothesized that leptin administration would lead to alteration in gut hormones that would promote further weight reduction and improve glucose homeostasis.

## 2. Materials and Methods

### 2.1. Study Subjects

Women between the ages of 25 and 65 years who were at least 18 months after RYGB, had a percent total weight loss from the highest presurgical weight to current weight between 18% and 45%, and had a current BMI of 28–50 kg/m^2^ were invited to participate. Subjects were considered for enrollment if their plasma leptin level was less than the level predicted from the regression equation generated using leptin levels and BMI from a non-weight-reduced cohort of 55 women who had participated in previous studies from our group: (0.991 × BMI) − 3.37. [JK, unpublished data]. Exclusion criteria have been described elsewhere [32]. This study is in accordance with the guidelines of the Declaration of Helsinki and was approved by the Columbia University Institutional Review Board. All subjects provided written informed consent.

Thirty-five of the 69 subjects screened met enrollment criteria. Eight subjects failed to have at least one follow-up visit after randomization and were excluded from the analysis. Of the remaining 27 subjects, 22 completed the test meal at baseline and 16 weeks after treatment [32].

### 2.2. Protocol

The study protocol has been described previously [32]. Briefly, subjects who met criteria entered a 2 week single-blind placebo run-in period, after which they were randomized to receive either placebo or recombinant human metreleptin. Metreleptin, referred to as “leptin,” and placebo were generously donated by Amylin Pharmaceuticals (San Diego, CA). The dose of leptin (0.05 mg/kg body weight self-administered via subcutaneous injection twice daily) was expected to raise maximum plasma leptin levels to high physiologic/low pharmacologic levels yet would not be expected to cause clinically significant weight loss in a person who had not undergone weight reduction [25].

At weeks 0 and 16, a meal challenge (Optifast; Novartis, Minneapolis MN; 474 mL, 320 Kcal, 50% carbohydrate, 35% protein, and 15% fat) was performed. PYY, insulin, glucose, insulin, C-peptide, and ghrelin (total, acyl, and desacyl) were measured in the fasted state as well as 15, 30, 60, 90, and 120 minutes after consumption of the liquid beverage, with the exception of total GLP-1 that was measured in the fasted state as well as 15 and 30 minutes postprandially. Appetite was assessed using a validated visual analog scale [33] with questions about a subjective feeling written below a 100 mm line anchored on either end with opposite descriptors (not at all, extremely). Subjects were asked to make a vertical mark across the line corresponding to their feelings. The answer was quantified by measuring the distance from the left end of the line to the mark. The VAS was administered in the fasted state as well as 60, 90, and 120 minutes after consumption of the liquid meal.

Venous blood samples were collected in EDTA tubes that were centrifuged for 15 minutes at 4°C and stored at −80°C until assayed in duplicate. Glucose, insulin, leptin, and total ghrelin were measured as described elsewhere [11]. Assays for total PYY and total GLP-1 were previously described as well [34]. C-peptide was measured with the Immulite Analyzer (Diagnostic Products Corp., Los Angeles, CA). Glucagon was measured by RIA as per manufacturer's instructions (Millipore Corporation, Billerica, MA). Blood samples for the measurement of acyl and desacyl-ghrelin were collected in EDTA tubes containing AEBSF, were centrifuged, and then acidified with HCl prior to measurement by sandwich assays [35]. Plasma was diluted as necessary to obtain readings within the assay range.

### 2.3. Statistical Analysis

Data were evaluated for normality with the Kolmogorov-Smirnov test and none were found to require transformation. Raw score differences and percent change from baseline were calculated. For repeatedly measured postprandial samples, area-under-the-curve was calculated using the trapezoidal rule. Group differences at baseline were evaluated with independent *t*-tests for continuous measures. Group, time, and group by time interactions were estimated with linear mixed models for repeated measures as fixed effects, the value of the outcome at baseline entered as a continuous covariate, and a compound symmetry covariance structure for the autocorrelation of measures within subject. The covariance structure was selected prior to inferential testing from empirical evaluation of alternative structures. Model estimated mean and standard errors for differences between times within group and between groups at specific times were used for the specific comparisons. *P* values for differences are based on the method of simultaneous confidence intervals. Least squares regression was used to assess the association of weight loss to initial leptin levels, number of months between surgery and study entry, and percent weight loss from presurgical maximum weight. No adjustment for multiplicity was employed for the multiple endpoints assessed.

## 3. Results

Prior to RYGB, BMI was similar between groups (Table 1). Baseline characteristics, with the exception of age, were also similar at the time of this study. Duration of postoperative period was a mean of 53 ± 2.3 months for the study cohort. Mean percent weight loss from the highest preoperative weight to weight at time of screening visit was 30.7 ± 0.33%, with a range of 18.2–44.7%. The mean BMI for the study cohort at the time of screening was 34.6 ± 0.2 kg/m^2^, with a range of 28.4–41.7 kg/m^2^.

**Figure 1:**
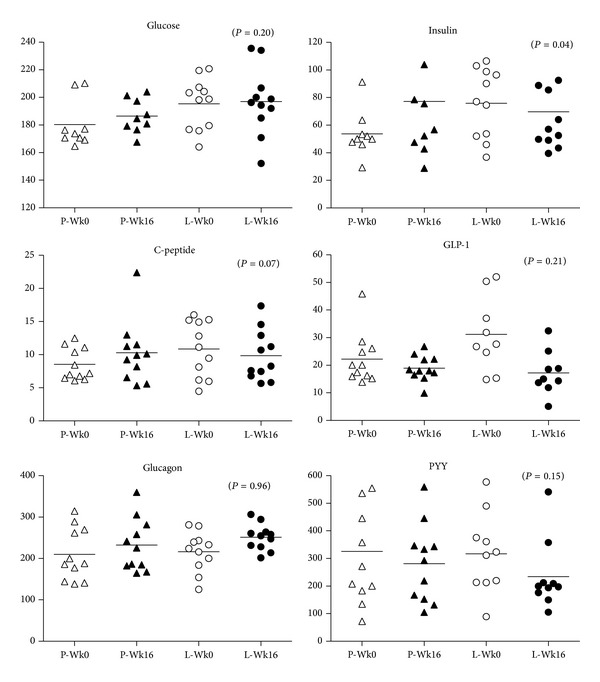
Levels of glucose, insulin, C-peptide, GLP-1, glucagon, and PYY. GLP-1 in placebo and leptin treated groups at weeks 0 and 16 (triangles, placebo group; circles, leptin group). Individual values and group mean (solid line) are represented.

**Figure 2:**
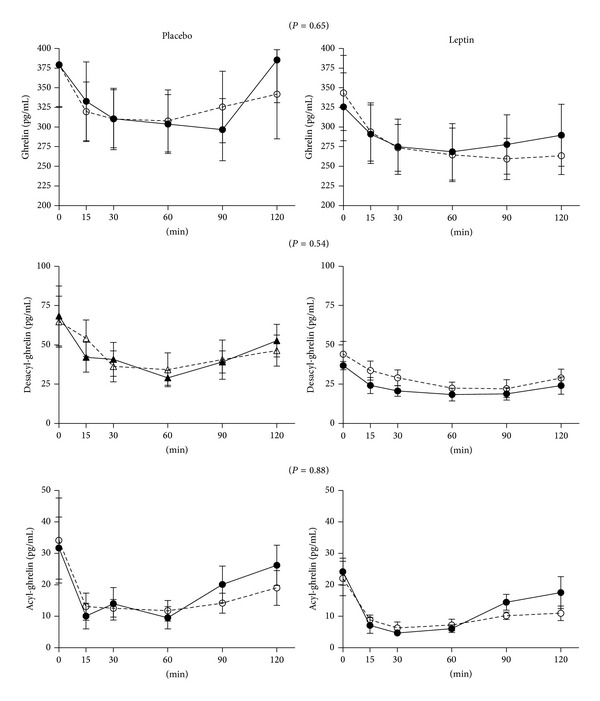
Fasting and postprandial plasma levels of total ghrelin, acyl-ghrelin, and desacyl-ghrelin (open circles and dashed line, week 0; closed circles and solid line, week 16).

At 16 weeks there was no significant change in weight within or between groups (Table 2). As expected, leptin concentrations increased in the treated group. Group by time interaction testing revealed significant differences between leptin and placebo treated groups for fasting PYY and insulin AUC (Table 2); however, the latter difference was driven by one subject who had an unusually elevated postprandial insulin and C-peptide response in the placebo group at week 16 (Figure 1). Otherwise, there were no changes in glucostatic parameters that were different between the groups. Fasting ghrelin and the ghrelin response to the test meal were similar between groups (Figure 2). Similarly, acyl-ghrelin, desacyl-ghrelin, and the ratio of desacyl-to acyl-ghrelin in the fasted and postprandial state did not change with leptin treatment.

One question on the VAS scale showed a statistically significant difference between the leptin and placebo treated groups at week 16 (*P* = 0.05; Table 3). “How much do you crave something sweet right now?” The leptin treated group had a significantly lower rating for this question than did the placebo treated group.

## 4. Discussion

Our previously published report of this investigation showed that physiological leptin replacement in weight stable women who are in a state of relative leptin insufficiency after RYGB does not result in further weight loss [32]. Absence of an effect on body weight allowed for the unique opportunity to examine the role of leptin on gut hormone and glucose regulation independent of weight loss. Our findings indicate that leptin replacement does not lead to alterations in gut hormone physiology that would favor a further reduction in weight or improvement in glucose homeostasis compared to placebo in this population of obese women after RYGB.

Ghrelin is an orexigenic hormone that has an opposing effect to the anorexigenic properties of leptin. Leptin has been found to decrease ghrelin release from the stomach* in vitro* and suppress ghrelin secretion from wild type and* ob/ob* mouse models as well as diabetic rat models [17, 18, 36]. However, this effect was observed only transiently in adult rats [37]. Ghrelin O-acyl transferase (GOAT), the enzyme that octanoylates ghrelin from the desacylated to the acylated form, is increased by leptin* in vitro* in stomach cells, thus increasing expression of the more potent acylated form and promoting food intake [38]. Desacyl-ghrelin has been shown in some, but not all, studies to favor insulin sensitivity and lack of weight gain [18, 36, 38]. Glucose-stimulated insulin secretion was observed in rats following central desacyl-ghrelin administration but not following peripheral administration [39]. Our findings showed no changes in ghrelin levels after leptin administration, suggesting that modulation of ghrelin levels or acylation may not be dependent upon leptin in the weight-reduced state or that our subjects were resistant to the effects of leptin.

Leptin and PYY both play roles in appetite reduction yet it is unclear whether they work synergistically or independently toward this common goal.* Ad libitum* fed rats administered PYY followed by leptin had prolonged satiation compared to when administered PYY alone [40], suggesting that the two hormones work synergistically. Leptin administration to humans following diet-induced weight loss did not increase PYY levels following a short term fast [40], which is similar to our findings in surgically induced weight loss, suggesting that PYY is not dependent on leptin to exert satiating effects.

Postprandial GLP-1 levels have been found to increase following RYGB [41] and may contribute to a reduction in appetite, weight loss, and improved glucose homeostasis. GLP-1 and leptin interact to cause a reduction in food intake in* ob/ob* mice and rat models, with GLP-1 secretion being enhanced following leptin administration [15, 16]. We did not observe an increase in GLP-1 levels after leptin treatment. Although AUC measurement of GLP-1 decreased after leptin treatment, which is contrary to animal models and counter to what we expected based on the role of GLP-1, the group by time interaction was not significant. A possible limitation of this study is that total, and not active, GLP-1 was measured.

Leptin, glucagon, and insulin work collaboratively to maintain normoglycemia through inhibitory and stimulatory effects on each other. The relationship between insulin and leptin has been heavily studied, with findings most recently suggesting that leptin has an inhibitory effect on insulin secretion and reduces glucagon secretion in rodents with type 1 diabetes, resulting in improved glycemic control in the absence of insulin [24]. Restoration of leptin receptors on proopiomelanocortin neurons in obese mice normalized blood glucose and ameliorated hepatic insulin resistance and hyperglucagonemia independent of changes in body weight [24]. In humans, leptin administration had a minimal impact on glycemic control (HbA1c decreased from 8.01% to 7.96%) in patients with type 2 diabetes who maintained a stable weight over the 4 month study period [42]. Similarly, leptin treatment did not have a clinically important effect on insulin action in obese people with newly diagnosed type 2 diabetes [43]. Our findings are in disagreement with mouse studies showing a reduction in glucagon secretion in the presence of leptin. A limitation of our study is that although subjects were obese, they were not hyperglycemic or insulin resistant as evaluated by HOMA-IR; thus, results may not be indicative of results that might be obtained using leptin therapy in a population with glucose intolerance or before diabetes. Also, the small study size would not be able to detect very small changes.

Feelings of hunger and satiety data were not different between groups with the exception of a decreased craving for something sweet in the leptin treated group at week 16 compared to placebo treated subjects at that time point. Leptin has been shown to modulate sweet sensitivity at the level of the taste receptors of the tongue.* Ob/ob* mice were found to have increased cravings for sweet items that were decreased following administration of leptin [44]. This did not occur in the leptin receptor deficient* db/db* mice. Similarly, obese humans were found to have obliterated diurnal variation in sweet cravings that are present in nonobese humans and to have a higher threshold for sensitivity to the effects of leptin on sweet cravings, thought to be due to a higher basal leptin level [45]. Our findings suggest that this threshold can be overcome with leptin administration; however, such decreases in cravings were unable to elicit significant alterations in weight or glucose homeostasis but might conceivably play a role in the maintenance of weight loss over time.

Our study subjects were still overweight or obese at the time of study entry, and while they had lost a considerable amount of weight and had leptin levels below those predicted for their current BMI, there is a possibility that they continued to be leptin resistant, manifesting no significant changes to gut related hormones or feelings of satiety and satiation. Our results are specific to a stable weight-reduced state and, thus, may not indicate whether leptin administration can modulate hormone levels during active weight loss. The question then becomes, at what level of leptin or at what weight or at what amount of weight loss would the resistance begin to subside, thus allowing those undergoing weight loss to experience the weight regulating benefits of leptin and possibly of appetitive hormones regulated by leptin. There may also be subpopulations, such as individuals with sweet cravings, who may benefit from longer exposure to exogenous leptin administration in order to help maintain a reduced body weight.

## 5. Conclusion

Leptin administration to women after RYGB who are in a state of relative leptin insufficiency does not lead to alterations in gut hormones or hormones that control glucose homeostasis in the absence of weight reduction, suggesting that the effects of these hormones to promote weight loss are not necessarily leptin-dependent. Sweet cravings were found to be decreased in those subjects receiving leptin, suggesting a possible therapeutic benefit of leptin administration in a subset of individuals.

## Figures and Tables

**Table 1:** Baseline characteristics of study participants.

Parameter	Placebo	Leptin	*P* value*
Age (y)	42.2 ± 2.8	51.4 ± 2.0	0.02
Pre-RYGB BMI (kg/m^2^)	48.6 ± 1.9	47.1 ± 1.8	0.58
BMI at screen (kg/m^2^)	35.0 ± 1.1	33.3 ± 1.4	0.35
Wt Loss (%)	30.2 ± 2.3	30.7 ± 2.1	0.88
Post-op period (mo)	44.2 ± 7.4	64.6 ± 8.6	0.09
Leptin (ng/mL)	27.1 ± 3.2	21.8 ± 2.5	0.20
Leptin/kg FM (ng/mL/kg)	0.70 ± 0.06^a^	0.66 ± 0.06^b^	0.61

Results are expressed as mean ± SEM. *n* = 11 subjects per group except as follows: ^a^ *n* = 9; ^b^ *n* = 10. *P value obtained by two-tailed independent *t*-test.

**Table 2:** Changes in weight, glucostatic, and appetitive hormones.

	Placebo		Leptin		P*
	Week 0	Week 16	Week 0	Week 16	
Wt (kg)	91.5 ± 4.9	91.1 ± 5.1	86.2 ± 3.8	86.4 ± 3.9	0.74
Leptin (ng/mL)	27.1 ± 3.2	29.8 ± 4.5	21.8 ± 2.5	223.4 ± 66^ab^	**0.01**
Glucose (mg/dL)	86.2 ± 1.8	84.4 ± 2.5	88.0 ± 1.7	88.0 ± 2.1	0.49
Insulin (*μ*IU/mL)	3.0 ± 0.4	3.6 ± 0.6	4.2 ± 0.7	4.1 ± 0.8	0.34
Glucose AUC	10354 ± 599	11506 ± 294	11718 ± 338^b^	11814 ± 438	0.20
Insulin AUC	2990 ± 327	4693 ± 888^a^	4553 ± 455	4181 ± 561	**0.04**
HOMA-IR	0.63 ± 0.08	0.76 ± 0.13	0.90 ± 0.14	0.89 ± 0.19	0.40
CPEP (ng/mL)	1.31 ± 0.11	1.30 ± 0.15	1.42 ± 0.14	1.40 ± 0.17	0.94
CPEP AUC	8.5 ± 0.7	10.3 ± 1.4	10.9 ± 1.3	9.8 ± 1.2	0.07
GLP-1 (pg/mL)	11.5 ± 2.1	9.7 ± 2.2	6.8 ± 1.6	7.1 ± 1.4	0.54
GLP-1 AUC	1332 ± 166	1136 ± 84	1957 ± 395	1171 ± 198^a^	0.21
PYY (pg/mL)	69.1 ± 12.6	43.0 ± 10.6^a^	47.9 ± 10.4	58.5 ± 15.6	**0.04**
PYY AUC	19536 ± 2483	16968 ± 2793	19557 ± 3405	21619 ± 3380	0.15
Glucagon (pg/mL)	79 ± 7	75 ± 7	71 ± 7	82 ± 5	0.10
Glucagon AUC	197 ± 20	231 ± 20^a^	216 ± 15	251 ± 10^a^	0.96
Ghrelin (pg/mL)	379 ± 54	379 ± 53	343 ± 48	326 ± 43	0.42
Acyl-ghrelin (pg/mL)	34.1 ± 13.5	31.7 ± 9.9	18.8 ± 5.4	21.9 ± 4.4	0.59
Desacyl-ghrelin (pg/mL)	64.7 ± 16.3	68.4 ± 19.0	41.4 ± 0.4	37.2 ± 3.2	0.63

Results are expressed as mean ± SEM. *n* = 11 subjects per group except for ghrelin, acyl-ghrelin, and desacyl-ghrelin, where *n* = 9 for placebo group. Hormone measurements are from the fasted state unless otherwise indicated. ^a^ *P* < 0.05; week 16 is statistically different from week 0 within group. ^b^ *P* < 0.05; difference in values is statistically significant between groups at the same week. *P < 0.05; statistically significant values for group by time interaction.

**Table 3:** Visual analog scale.

	Placebo		Leptin	
	Week 0	Week 16	Week 0	Week 16
How hungry are you?	2460 ± 479	3761 ± 569	2747 ± 524	3200 ± 592
How satisfied are you?	7606 ± 819	6537 ± 924	5283 ± 885	4719 ± 969
How full do you feel?	7428 ± 785	6241 ± 890	5286 ± 848	5197 ± 933
How much do you crave something sweet?	2120 ± 517	2898 ± 563*	2391 ± 543	1461 ± 585*
How much can you eat now?	3221 ± 454	4264 ± 529	3453 ± 492	3246 ± 557
How much nausea or discomfort do you feel?	2958 ± 508	2330 ± 577	3056 ± 549	2888 ± 604

Values represent mean ± SEM AUC from fasting to 120 min after consumption of the test meal. *P < 0.05 for within-group change.
